# The small heat shock protein B8 (HSPB8) modulates proliferation and migration of breast cancer cells

**DOI:** 10.18632/oncotarget.14422

**Published:** 2017-01-02

**Authors:** Margherita Piccolella, Valeria Crippa, Riccardo Cristofani, Paola Rusmini, Mariarita Galbiati, Maria Elena Cicardi, Marco Meroni, Nicola Ferri, Federica F. Morelli, Serena Carra, Elio Messi, Angelo Poletti

**Affiliations:** ^1^ Dipartimento di Scienze Farmacologiche e Biomolecolari (DiSFeB), Centro di Eccellenza sulle Malattie Neurodegenerative, Università degli Studi di Milano, Milano, Italy; ^2^ C. Mondino National Neurological Institute, Pavia, Italy; ^3^ Dipartimento di Scienze del Farmaco, Università degli Studi di Padova, Padova, Italy; ^4^ Dipartimento di Scienze Biomediche, Metaboliche e Neuroscienze, Università di Modena e Reggio Emilia, Modena, Italy

**Keywords:** breast cancer, HSPB8, proliferation, migration, MCF-7 cells

## Abstract

Breast cancer (BC) is one of the major causes of cancer death in women and is closely related to hormonal dysregulation. Estrogen receptor (ER)-positive BCs are generally treated with anti hormone therapy using antiestrogens or aromatase inhibitors. However, BC cells may become resistant to endocrine therapy, a process facilitated by autophagy, which may either promote or suppress tumor expansion. The autophagy facilitator HSPB8 has been found overexpressed in some BC. Here we found that HSPB8 is highly expressed and differentially modulated by natural or synthetic selective ER modulators (SERMs), in the triple-positive hormone-sensitive BC (MCF-7) cells, but not in triple-negative MDA-MB-231 BC cells. Specific SERMs induced MCF-7 cells proliferation in a HSPB8 dependent manner whereas, did not modify MDA-MB-231 cell growth. ER expression was unaffected in HSPB8-depleted MCF-7 cells. HSPB8 over-expression did not alter the distribution of MCF-7 cells in the various phases of the cell cycle. Conversely and intriguingly, HSPB8 downregulation resulted in an increased number of cells resting in the G0/G1 phase, thus possibly reducing the ability of the cells to pass through the restriction point. In addition, HSPB8 downregulation reduced the migratory ability of MCF-7 cells. None of these modifications were observed, when another small HSP (HSPB1), also expressed in MCF-7 cells, was downregulated. In conclusion, our data suggest that HSPB8 is involved in the mechanisms that regulate cell cycle and cell migration in MCF-7 cells.

## INTRODUCTION

Breast cancer (BC) is the most frequent cause of cancer death in women in underdeveloped countries, while in western countries is the second cause of death, after lung cancer [1]. About 70% of cases positive for estrogen receptors (ERs) expression, and patients with ER-positive BC generally receive anti-hormonal therapy, mainly based on anti-estrogens, selective ER modulators (SERMs), or inhibitors of aromatase, which sinthesizes estrogens. The SERMs are natural or synthetic compounds which bind ERs and display tissue specific mixed agonistic-antagonistic activities [2]. Some SERMs have been approved for BC treatment (e.g.: tamoxifen, raloxifen) [3], while others, such as some natural phytoestrogens, might have a chemo preventive activity [4], an aspect still largely debated [5, 6]. The SERMs mechanism of action depends on three interdependent factors: the level of ER isoform (ERα or ERβ) expression in a given target tissue, the different conformational changes of ERs induced by a given SERM and the binding of transcriptional regulatory proteins to ERs. In several cases, BC cells become resistant to these anti-hormonal therapies [7], giving rise to more aggressive tumors (e.g. tamoxifen-resistant BC). It has been recently proposed that the mechanism responsible for anti-hormonal resistance in BC is facilitated by autophagy [8], a process responsible for the degradation of damaged proteins or organelles [9]. In cancer cells, autophagy may have a suppressive activity in the initial phase of the tumor growth, while in later stages, autophagy may enhance cancer cells survival. In several tumors, autophagy involves the small heat shock proteins HSPB8 (also known as HSP22, E2IG1, small stress protein 1 or H11), an autophagic flux enhancer that acts in conjunction with BAG3 [10–13], HSPA8/HSC70 and STUB1/CHIP to target cargoes to autophagosomes (Cristofani et al., in press; [14, 15]), and their recognition by SQSTM1/p62 [14]. In stressful conditions (e.g.: hypoxia or nutrient deprivation), HSPB8 is upregulated [16], and by facilitating autophagy, mediates amino acids recycle enhancing cancer cell capability to survive under unfavorable conditions. HSPB8-BAG3 complex also recognizes damaged cytoskeletal proteins, determining their selective degradation by autophagy [17]. Moreover, the complex regulates actin dynamic modulation during mitosis by influencing spindle orientation required for chromosomes alignment at the metaphase plate and chromosome segregation [18]. HSPB8 or BAG3 silencing results in disorganization of actin-rich retraction fibers and alters spindle orientation: HSPB8-BAG3 complex may thus mediated quality control mechanism during mitotic processes activated in proliferating cells [18].

HSPB8 activity in cancer cells is rather complex. HSPB8 is highly expressed in non-endocrine tumors [19, 20], in which it generally reduces cell proliferation and/or induces apoptosis. In other tumors (melanomas, prostate cancer (PC), Ewing’s sarcoma and leukemia), HSPB8 gene is methylated and poorly expressed [21, 22]. Using gene array on laser capture microdissection (LCM) of tumors from transgenic erbB2, ras, and cyclin D1 mice, HSPB8 expression was found to be higher in invasive lesions than in preinvasive lesions [23]. A similar pattern was observed in LCM from human ductal carcinoma [23]. When exogenously expressed in these tumors, HSPB8 activates caspase-3 cleavage and apoptosis [19, 22]. Surprisingly, in other tumor types HSPB8 expression correlates with increased aggressiveness [24, 25]. HSPB8 is highly expressed in BC tumors, particularly in the ER-positive tumors [26, 27], and in cell lines of hormone-resistant BC, suggesting its involvement in the acquisition of resistance to hormone therapy [28]. HSPB8 is induced by estrogens and cadmium (a metalloestrogen) in the ER-positive BC MCF-7 cells [25], a process reverted by the pure anti-estrogen ICI182.780 (faslodex, fulvestrant). Instead, the SERM tamoxifen had no effects on HSPB8 [25], but HSPB8 is highly overexpressed in tamoxifen-resistant BC (MCF-7) cell subclones [28], possibly favouring appearance of drug-resistance in cancer cells [28, 29]. Similarly, in multiple myeloma (MM), the chemotherapic bortezomib (Velcade, a UPS inhibitor) induces the development of bortezomib-resistant cells, in which HSPB8 is robustly overexpressed [24].

Here, we evaluated the role of HSPB8 in proliferation, cell cycle modification and cell migration of cultured human BC cells, and its possible modulation by selected SERMs. We also analysed the effects of either overexpression or downregulation of HSPB8 during concomitant treatments with different SERMs. We found that the increased proliferation induced by all SERMs was reduced by the concomitant downregulation of HSPB8, which led to cell cycle arrest in the G0/G1 phase and reduced the migratory ability of MCF-7 cells.

## RESULTS

### Expression of HSPB8 in different cell types

**Figure 1:**
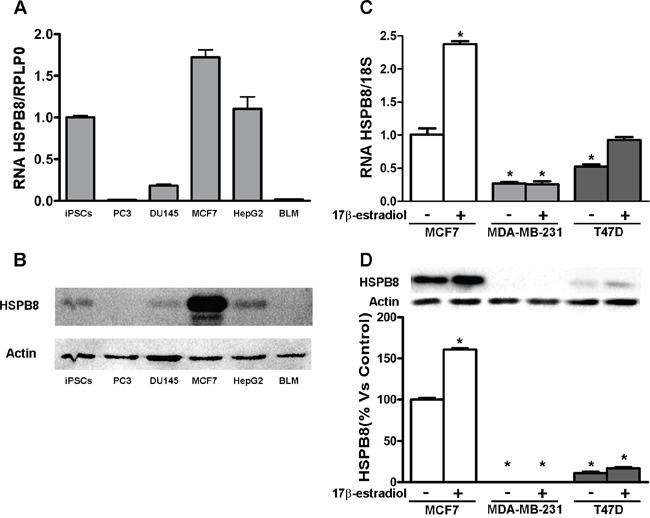
Expression of HSPB8 in different human cell lines HSPB8 mRNA and protein levels were measured by real-time RT-PCR analysis **A**. and Western blot analysis **B**. in iPSCs, PC3, DU145, MCF-7, HepG2 and BLM cell lines. iPSCs: induced pluripotent stem cells; PC3 and DU145: prostate cancer cells; MCF-7: breast cancer cells; HepG2: hepatocellular carcinoma; BLM: melanoma cells. HSPB8 mRNA and protein levels were measured by real-time RT-PCR analysis **C**. and Western blot analysis **D**. in three different human breast cancer cell lines treated or not for two days with 17β-estradiol (10nM): MCF7; MDA-MB-231 and T47D. *p<0.05 *vs* MCF-7 cells without treatment (first column). Values represent the mean from three independent experiments.

To assess the potential role of HSPB8 in BC cells, we initially compared its expression level in MCF-7 cells with that of other highly proliferating tumor cells and with undifferentiated induced pluripotent stem cells (iPCSs), maintaining cells plated in basal growth condition for 4 days, before they reached confluency. Expression analysis performed using real-time RT-PCR showed that HSPB8 mRNA is highly expressed in iPSCs, in BC cells (MCF-7) and in hepatocellular carcinoma (HepG2), while its expression is much lower in prostate cancer (PC) cells (PC3 and DU145 cells) and in melanoma cells (BML) (Figure 1, panel A). When HSPB8 protein levels were quantified in Western blot analysis (Figure 1, panel B), we found that MCF-7 cells were characterized by very high content of HSPB8 as compared to the other cell clones selected. We next evaluated HSPB8 expression in other BC cell lines and its possible modulation by 17β-estradiol. By analysing MCF-7, MDA-MB-231 and T47D BC cell lines, we found that in T47D cells HSPB8 is expressed at lower levels compared to MCF-7 cells; HSPB8 is poorly expressed in MDA-MB-231. 17β-estradiol further increased HSPB8 mRNA expression levels in MCF-7 and T47D cells, but not in MDA-MB-231 cell line (Figure 1, panel C). These data were also confirmed at protein levels (Figure 1, panel D).

### Effects of SERMs on MCF-7 and MDA-MB-231 cell growth

**Figure 2:**
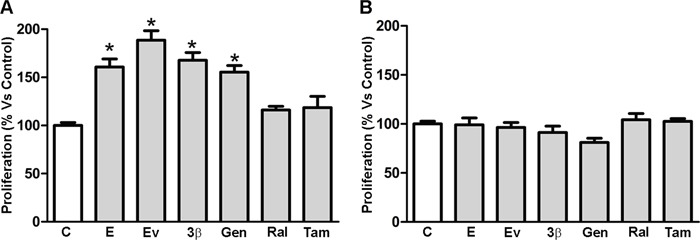
Cellular proliferation of MCF-7 and MDA-MB-231 cell lines. MCF-7 **A**. and MDA-MB-231 **B**. cellular proliferation was evaluated by MTT assay 2 days after treatment with DMSO (Control), 17β-estradiol (10nM), estradiol valerate (10nM), 3β-Adiol (1μM), genistein (1μM), raloxifen (1μM) and tamoxifen (1μM). Statistical analysis was performed by one-way ANOVA followed by Bonferroni multiple comparison tests. *p<0.05 *vs* Control. Values represent the mean from three independent experiments. C. Control cells; E: 17β-estradiol; EV: estradiol valerate; 3β: 3β-Adiol; Gen: genistein; Ral: raloxifen; Tam: tamoxifen.

We selected specific estrogens and SERMs to evaluate their capability to modulate MCF-7 and MDA-MB-231 cell proliferation, under growing conditions. We used 17β-estradiol and estradiol valerate at 10nM doses, 3β-Adiol, the natural phytoestrogen genistein, raloxifen and tamoxifen at 1μM concentrations. We thus performed a MTT assay to measure MCF-7 and MDA-MB-231 cell proliferation/viability. Growth analysis revealed that proliferation of MCF-7 cells was significantly increased after 2 days of treatment with all estrogenic compounds tested, including genistein (Figure 2, panel A). The most potent activity was associated to estradiol valerate, which almost doubled the proliferation/viability of MCF-7 cells (Figure 2, panel A). As expected, both raloxifen and tamoxifen, used as controls, were unable to modify the proliferation/viability rate of MCF-7 cells (Figure 2, panel A). On the contrary, 2 days treatment with all the considered SERMs did not modify MDA-MB-231 cell growth (Figure 2, panel B).

### Effects of SERMs on the endogenous HSPB8 expression in MCF-7 cells

**Figure 3:**
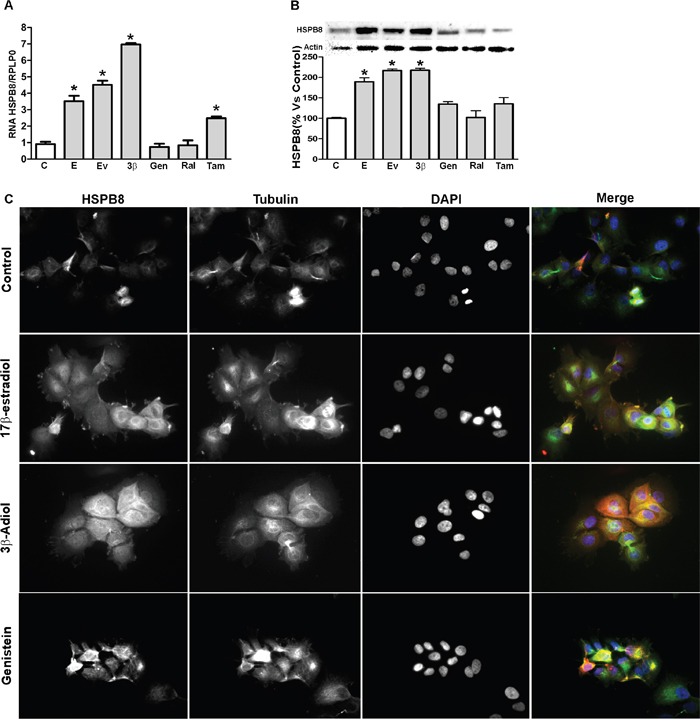
Expression of HSPB8 in MCF-7 cell line HSPB8 mRNA and protein levels were quantified by real-time RT-PCR analysis. **A**. and Western blot analysis **B**. 2 days after treatment with DMSO (Control), 17β-estradiol (10nM), estradiol valerate (10nM), 3β-Adiol (1μM), genistein (1μM), raloxifen (1μM) and tamoxifen (1μM). Statistical analysis was performed by one-way ANOVA followed by Bonferroni multiple comparison tests. Representative pictures of immunofluorescence staining of HSPB8 (red, anti-rabbit) and α-tubulin (green, anti-mouse) in MCF-7 cells, treated as above for 2 days. DAPI (blue) was used to stain DNA **C**. *p<0.05 *vs* Control. Values represent the mean from three independent experiments. C. Control cells; E: 17β-estradiol; EV: estradiol valerate; 3β: 3β-Adiol; Gen: genistein; Ral: raloxifen; Tam: tamoxifen.

We next evaluated whether the drugs could also further increase the already high levels of HSPB8 in MCF-7 cells. HSPB8 mRNA and protein levels were analysed in MCF-7 cells treated for 48 hrs with selected active dose (based for each compound on their relative Kd for ERs). In particular, we used 17β-estradiol and estradiol valerate at 10nM concentrations, 3β-Adiol, genistein, raloxifen and tamoxifen at 1μM concentrations. HSPB8 mRNA evaluated in real-time RT-PCR analysis (Figure 3, panel A) demonstrated that both estradiol (and its valerate form, which both bind equally the two ERs [5, 30–34]) and 3β-Adiol (which binds preferentially ERβ exerting agonistic activity) [6, 35] were able to induce a robust increase of HSPB8 expression in MCF-7 cells. Surprisingly, genistein, which acts as a natural SERM (with ERβ preferential binding and agonistic activities [36]) did not significantly modify HSPB8 expression. The synthetic SERM raloxifene (characterized by a poor antiestrogenic activity) was also unable to induce HSPB8 expression, while, the other synthetic SERM selected, tamoxifen (which is considered a potent ER antagonist in BC cells) induced two-fold HSPB8 expression (Figure 3, panel A). Similar results were observed at protein levels. In fact, Western blot analysis (Figure 3, panel B) showed that HSPB8 protein levels are increased by the treatment with estradiol (and its valerate form) and by 3β-Adiol. All SERMs (natural or synthetic, including tamoxifen) were unable to alter HSPB8 protein levels in MCF-7 cells. The induction of HSPB8 mRNA and protein levels observed using real-time RT-PCR and Western blot analyses were further confirmed by immunofluorescence analysis on MCF-7 cells treated with 17β-estradiol and 3β-Adiol. An intense increase of HSPB8 immunoreactivity was found after exposure to 17β-estradiol, 3β-Adiol; a slight increase was observed in cells treated with genistein (Figure 3, panel C).

### HSPB8 silencing in MCF-7 cell line and its effect on ERα and ERβ expression

**Figure 4:**
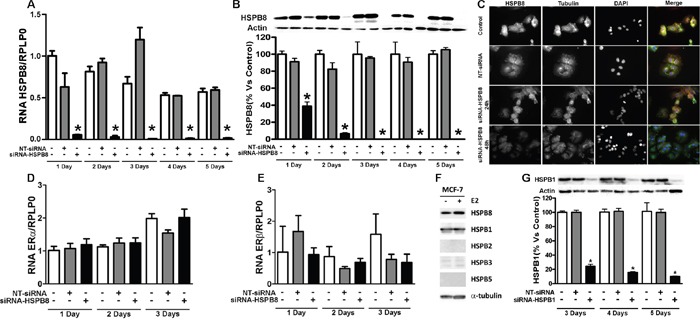
HSPB8 silencing and its effect on expression of ERα and ERβin MCF-7 cell line MCF-7 cells were transfected with siRNA-HSPB8 or NT-siRNA and the analysis was performed 1, 2, 3, 4 and 5 days after transfection. The HSPB8 mRNA and protein levels were measured by real-time RT-PCR **A**. and Western blot analysis **B**. Statistical analysis was performed by one-way ANOVA followed by Bonferroni multiple comparison tests *p<0.05 *vs* Control-1day (untransfected cells). Representative pictures of immunofluorescence staining of HSPB8 (red, anti-rabbit) and α-tubulin (green, anti-mouse) in MCF-7 cells transfected for 1 and 2 days. DAPI (blue) was used to stain DNA **C**. NT = Non-targeting. ERα **D**. and ERβ **E**. mRNA levels were measured by real-time RT-PCR analysis 1, 2, and 3 days after transfection. Values represent the mean from three independent experiments, no statistical differences were observed. The expression of different small heat shock protein was evaluated by Western blot analysis in MCF-7 cell line F. MCF-7 cells were transfected with siRNA-HSPB1 or NT-siRNA and HSPB1 protein levels were measured by Western blot analysis 3, 4 and 5 days after transfection **G**. Statistical analysis was performed by one-way ANOVA followed by Bonferroni multiple comparison tests *p<0.05 *vs* Control-1day (untransfected cells).

In order to evaluate whether HSPB8 is essential for BC cells dynamics, we designed specific siRNA to silence the expression of HSPB8. To produce and characterize HSPB8 knock-down cell models, MCF-7 cells were transfected with the designed siRNA-HSPB8 and analysed at different times (from 1 up to 5 days) after transfection. Using RealTime RT-PCR, we first evaluated HSPB8 mRNA levels. Figure 4, panel A shows that a significant reduction of HSPB8 mRNA levels was already detectable 1 day after transfection; HSPB8 silencing was complete 3 days lasting up to 5 days after transfection. When we analysed HSPB8 protein levels in Western blot analysis (Figure 4, panel B) we observed an identical trend of reduction to that found for mRNA levels, proving that HSPB8 silencing was very efficient in MCF-7 cells with the selected siRNA. The downregulation of HSPB8 mRNA and protein levels observed by RealTime RT-PCR and Western blot analyses (Figure 4, panel A and B) were further confirmed by immunofluorescence analysis using a specific anti-HSPB8 antibody. In fact, in MCF-7 cells treated with siRNA-HSPB8, we observed an almost complete disappearance of HSPB8 immunoreactivity after 48 hours of siRNA treatment (Figure 4, panel C).

To assess whether HSPB8 silencing was able to alter estrogenic responsiveness of MCF-7 cells, we analysed ERα and ERβ mRNA levels 1, 2, and 3 days after transfection of siRNA-HSPB8. Neither the expression of ERα (Figure 4, panel D), nor that of ERβ (Figure 4, panel E) were affected by HSPB8 silencing. However, while the levels of ERα remained very similar in all samples analysed, we observed large variations of ERβ expression, even if these modifications never reached statistical significance. These alterations could be due to a rather marked fluctuation of the ERβ levels in MCF-7 cells in basal conditions.

To assess whether other HSPBs were expressed in MCF-7 cells, we initially quantified by Western blot analysis the protein levels of four different small heath shock proteins: HSPB1, HSPB2, HSPB3 and HSPB5. We found that MCF-7 cells expressed high levels of HSPB1, while HSPB2, HSPB3 and HSPB5 were not detectable (Figure 4, panel F). In order to compare the activity of these two HSPBs expressed in BC cells, we analysed whether HSPB1 downregulation recapitulates the modification we described after HSPB8 silencing: To this aim, we produced a siRNA directed against HSPB1 and transfected MCF-7 cells to evaluate HSPB1 protein levels. We found that HSPB1 protein levels were significantly reduced 3 days after transfection (Figure 4, panel G); and totally abolished 5 days after transfection, proving that HSPB1 silencing was very efficient in MCF-7 cells with the selected siRNA.

### Effects of HSPBs silencing on MCF-7 cell cycle progression

**Figure 5:**
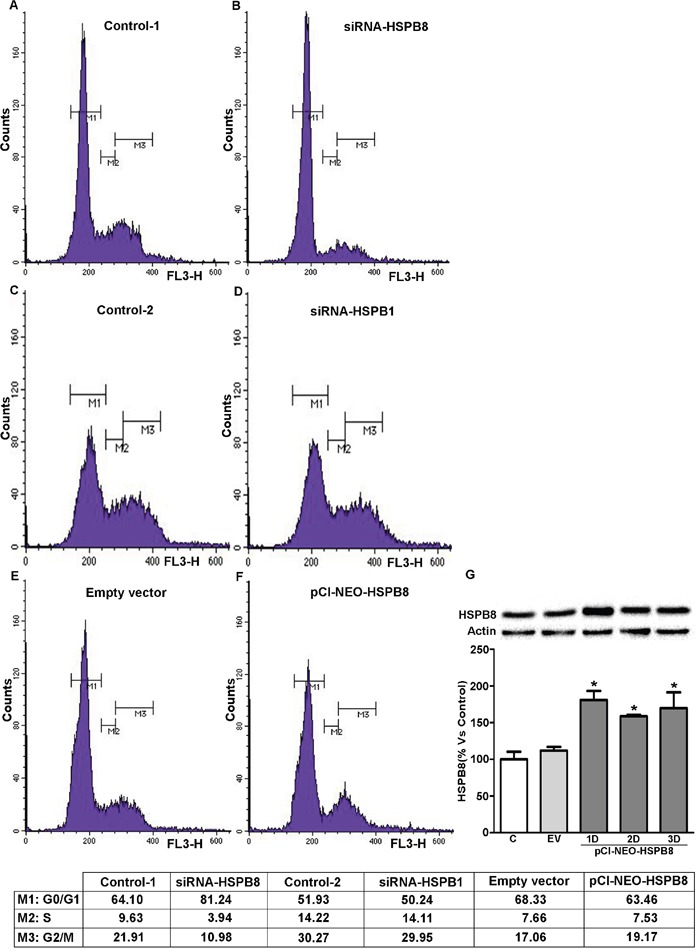
FACScan analysis of MCF-7 cells The adherent MCF-7 cells were treated with transfection medium (Control-1 and -2) **A-C**. transfected with siRNA-HSPB8 **B**., with siRNA-HSPB1 **D**., with empty vector as control **E**. and with plasmid pCI-NEO-HSPB8 that contains the sequence of human HSPB8 cloned in pCI **F**. After 3 days, the cells were measured on a FACScan flow cytometer with an argon laser at 488 nm for excitation and analyzed using Cell Quest software. Each experiment was performed in triplicate. The efficiency of HSPB8 overexpression in MCF-7 cells was evaluated by Western blot analysis, 1, 2 and 3 days after transfection **G**. Statistical analysis was performed by one-way ANOVA followed by Bonferroni multiple comparison tests *p<0.05 *vs* Control-1day (untransfected cells); EV empty vector.

On the basis of these data and on the recently described involvement of HSPB8 in the control of spindle orientation during mitosis [18], we next analysed if HSPB8 and HSPB1 silencing in MCF-7 could affect the cell cycle progression. We depleted HSPB8 and HSPB1 by siRNA transfection and, three days after transfection, we analyzed the distribution of MCF-7 cells through the cell cycle by FACS analysis. HSPB8 silencing correlated with a considerable increase of cell number resting in the G0/G1 phase (control, 64.10%; siRNA-HSPB8 81.24%), while the number of cells progressing in the S phase was remarkably decreased (control, 9.63%; siRNA-HSPB8 3.94%). In the G2/M cell cycle phase the number of HSPB8 silenced cells was diminished about 50% (control, 21.91%; siRNA-HSPB8, 10.98) (Figure 5, panels A and B). On the contrary, HSPB1 silencing did not modify neither the number of cells resting in the G0/G1 phase (control, 51.93%; siRNA-HSPB1 50.24%), nor the number of cells progressing in the S phase (control, 14.22%; siRNA-HSPB1 14.11%). In the G2/M cell cycle phase the number of HSPB1 silenced cells was slightly diminished (control, 30.27%; siRNA-HSPB1, 29.95%) (Figure 5, panel C and D).

To overexpress HSPB8, MCF-7 cells were transfected with control vector or with pCI-NEO-HSPB8 plasmid and analysed three days after transfection. The results indicated that HSPB8 overexpression correlated with a small reduction in the number of cells resting in G0/G1 phase (pCI-NEO-HSPB8, 63.64%; empty vector, 68.33%), while the population of cells progressing at the S phase was essentially unchanged (pCI-NEO-HSPB8, 7.53%; empty vector, 7.66%). Furthermore, HSPB8 overexpression induced a small increase in the number of cells accumulated in G2/M phases (19.17%) when compared to control cells (17.06%) (Figure 5, panel E and F). These data suggest that HSPB8 silencing induced cell cycle arrest in the G0/G1 phase of MCF-7 cells, while its overexpression did not alter their cell cycle distribution. Furthermore, to demonstrate overexpression efficiency of HSPB8, we transfected MCF-7 cells with control vector or pCI-NEO-HSPB8 plasmid to enhance HSPB8 levels at different times (from 1 up to 3 days) after transfection. Using Western blot analysis, we observed that there was a significantly increase of HSPB8 protein levels one day after transfection (Figure 5, panel G). HSPB8 protein levels remain elevated up to 3 days after transfection, proving that HSPB8 overexpression was very efficient in MCF-7 cells.

### Effects of HSPB8 overexpression on MDA-MB-231 cell cycle progression

**Figure 6:**
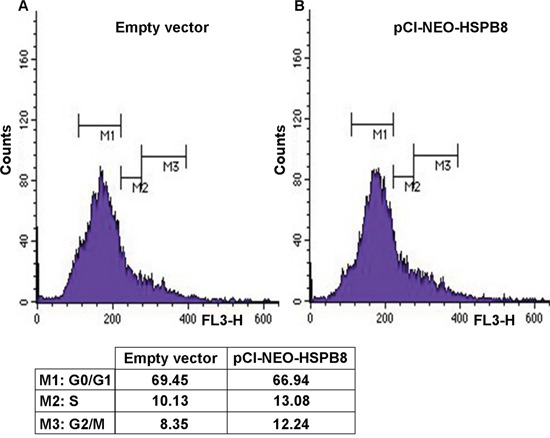
FACScan analysis of MDA-MB-231 cells MDA-MB-231 cells were transfected with empty vector as control **A**. and with plasmid pCI-NEO-HSPB8 that contains the sequence of human HSPB8 cloned in pCI **B**. After 3 days, the cells were measured on a FACScan flow cytometer with an argon laser at 488 nm for excitation and analyzed using Cell Quest software.

We next analysed the effects of HSPB8 overexpression in MDA-MB-231 cell cycle progression. We observed that HSPB8 overexpression in MDA-MB-231 cells correlated with a small decrease in the number of cells resting in G0/G1 phase (pCI-NEO-HSPB8, 66.94%; empty vector, 69.45%); in the same way, the population of cells progressing at the S phase was slightly increased (pCI-NEO-HSPB8, 13.08%; empty vector, 10.13%). Furthermore, HSPB8 overexpression induced a weakly increase in the number of cells accumulated in G2/M phases (12.24%) when compared to control cells (8.35%) (Figure 6, panel A and B). These data suggest that HSPB8 overexpression did not alter the MDA-MB-231 cell cycle distribution.

### Effects of HSPBs silencing and SERMs treatment on MCF-7 cell growth and migration

**Figure 7:**
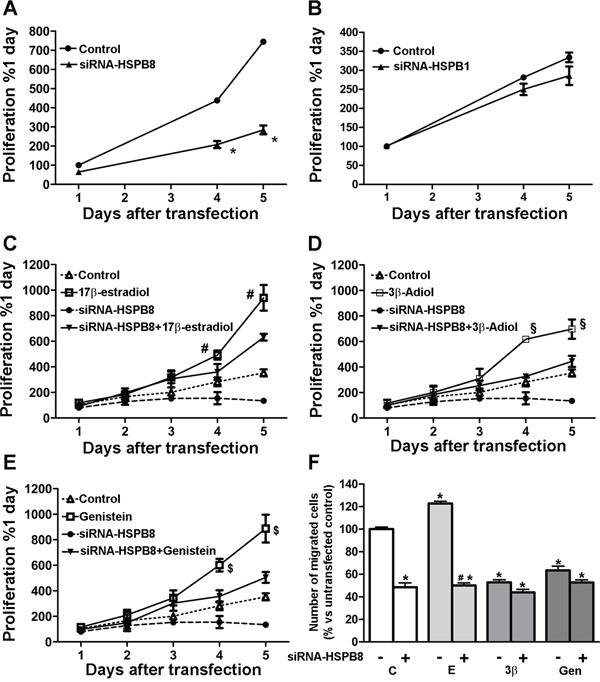
MCF-7 cells proliferation and migration MCF-7 cells were transfected with siRNA-HSPB8 A. or siRNA-HSPB1 B. and cellular proliferation was evaluated by MTT assay 1, 4 and 5 days after transfection. MCF-7 cells were transfected with siRNA-HSPB8 and treated for 5 days after transfection with 10nM 17β-estradiol **C**. 1μM 3β-Adiol **D**. and 1μM genistein **E**. Control: cells untransfected and treated with DMSO. Cellular proliferation was evaluated 1, 2, 3, 4 and 5 days after transfection. Statistical analysis was performed by two-way ANOVA followed by Bonferroni multiple comparison tests. Values represent the mean from three independent experiments. *p<0.01*vs* Control; #p<0.01 17β-estradiol vs siRNA-HSPB8+17β-estradiol; §p<0.01 3β-Adiol vs siRNA-HSPB8+3β-Adiol; $p<0.01 genistein vs siRNA-HSPB8+genistein. For the migration assay, MCF-7 untransfected cells were treated for 2 days with DMSO (first column), while the cells transfected with siRNA-HSPB8 were treated for 2 days after transfection with DMSO (second column), or with 17β-estradiol (E, 10nM), 3β-Adiol (3β, 1μM) and genistein (Gen, 1μM). Cells were then detached, and transferred to Boyden’s Chamber **F**. Statistical analysis was performed by one-way ANOVA followed by Bonferroni multiple comparison tests. Values represent the mean from three independent experiments. *p<0.05 *vs* untransfected cells (first column); #p<0.05 *vs* 17β-estradiol.

In order to study the role of HSPB8 and HSPB1 in the regulation of MCF-7 cell growth, we evaluated cellular proliferation by MTT assay 1, 4 and 5 days after HSPB8 and HSPB1 silencing. As shown in Figure 7, panel A, HSPB8 silencing correlated with a robust and significant decrease of MCF-7 cell growth when compared to control cells, while HSPB1 silencing did not alter MCF-7 cellular proliferation (Figure 7, panel B).

Next, MCF-7 cells were transfected or not with siRNA-HSPB8 and simultaneously treated for 2 days with vehicle (DMSO) or with 17β-estradiol, 3β-Adiol and genistein. Figure 7, panel C, shows that, as expected, 17β-estradiol induced a significant increase of MCF-7 cellular proliferation, but this estrogenic activity was significantly reduced 4 and 5 days after HSPB8 silencing. In the same manner, the growth of MCF-7 cells was significantly increased after 2 days of treatment with 3β-Adiol with respect to control cells (Figure 7, panel D), and this pro-proliferative effect appeared to be significantly diminished following HSPB8 silencing. Further, exposure to 1μM genistein increased MCF-7 cell proliferation, with a significant gain in the number of cells compared to untreated cells (Figure 7, panel E). Even in this case, the increased proliferation induced by genistein was reduced after HSPB8 silencing. These results suggest that the presence of HSPB8 is required for a proper estrogen-induced MCF-7 cell proliferation.

Then, we studied the impact of HSPB8 depletion on cell migration. Haptotaxis was evaluated in a Boyden’s chamber using laminin coated membranes, because preliminary experiments had indicated that MCF-7 cells fail to migrate in the absence of a specific substrate (data not shown). MCF7 cells were transfected or not with siRNA-HSPB8 and simultaneously treated for 2 days with vehicle (DMSO) or with 17β-estradiol, 3β-Adiol and genistein. HSPB8 silencing significantly reduced the percentage of migrating cells as compared to non-silenced cells (Figure 7, panel F). 17β-estradiol significantly increased the percentage of migrating cells with respect to control cells, indicating that 17β-estradiol has a pro-migratory activity on MCF-7 cells; this pro-migratory effect was significantly reduced by HSPB8 silencing (Figure 7, panel F). On the contrary, 3β-Adiol significantly reduced the percentage of migrating cells compared to DMSO-treated cells, indicating that, despite of being an estrogenic compound, 3β-Adiol has anti-migratory effect on MCF-7 cells, and it may be a physiological antagonist of 17β-estradiol. In the same manner, the exposure to genistein inhibited the migratory activity of MCF-7 cells; thus, like 3β-Adiol, also these natural SERMs has an opposite effect than 17β-estradiol on MCF-7 cell migration, which is not influenced by HSPB8 silencing.

Interestingly, in BC cells, autophagy is modulated through cell cycle arrest [37]. Moreover, interplay between autophagy and cell cycle regulation in the inhibition of cancer cell proliferation has been well documented [38]. We thus investigated whether changes in the expression levels of HSPB8, which affects cell cycle in MCF-7 cells, also alter autophagy. As shown in Supplementary Figure 1 there were no variations in different autophagy markers (LC3, SQSTM1/p62, TFEB and ZK-SCAN3).

## DISCUSSION

BC is a very common and heterogeneous disease, which too often becomes resistant to endocrine therapy. The mechanisms behind this resistance to treatment are still poorly understood. The enhanced capability of cancer cells to respond to different types of cell stress (or stress tolerance) is emerging as one of the factors that may confer resistance to chemotherapy [28, 29]. Stress tolerance is generally mediated by an increased function of the PQC system, and particularly of selected chaperones and of autophagy [28]. The chaperone HSPB8 enhances autophagic flux, thereby acting as a potent facilitator of the autophagic process [14, 15, 39, 40], and it has been found increased in tamoxifen-resistant BC cells [28].

Here, we analysed the involvement of HSPB8 in BC cell dynamics. We initially characterized human BC cells and found that triple-positive hormone-sensitive MCF-7 cells (but not triple negative MDA-MB-231) express very high mRNA and protein levels of HSPB8. MCF-7 cells also express HSPB1, but not HSPB2, HSPB3 or HSPB5. We then evaluated the effects of SERMs on HSPB8 expression and possible modulation of cell viability. We found a significant increase of MCF-7 cells viability exposed to estrogenic (particularly estradiol valerate) compounds, while the SERMs tamoxifen and raloxifen did not modify cell viability. Interestingly, genistein treatment resulted in a proliferative rather than anti-proliferative effect on MCF-7 cells. In agreement with previous data [23, 25], estradiol enhanced HSPB8 expression. Moreover, we found that some SERMs (synthetic like tamoxifen, and natural like 3β-Adiol), but not all SERMs (synthetic like raloxifen, and natural like genistein) induced HSPB8 mRNA and protein. Interestingly, HSPB8 silencing in MCF-7 cells did not affect ERs expression (neither ERα nor ERβ).

Since HSPB8 controls spindle orientation during mitosis [18], thus affecting cell cycle progression, we analyzed whether modulation of HSPB8 in MCF-7 would influence the cell cycle. We found that while HSPB8 over-expression did not modify the distribution of MCF-7 cells in the various phases of the cell cycle, HSPB8 silencing correlated with an increased number of cells resting in the G0/G1 phase, thus possibly reducing the ability of the cells to pass through the restriction point. These effects were specific for HSPB8, since HSPB1 downregulation did not alter cell distribution in the various phases of the cell cycle. These data extend some previous reports [23, 41] that proposed HSPB8 as a target associated with ER-positive tumors and cyclin D1, one of the key regulators of the cell cycle. In addition, we found that HSPB8 (but not HSPB1) silencing resulted in a great reduction of MCF-7 cell proliferation, even in presence of 17β-estradiol, which is a potent inducer of MCF-7 cellular proliferation. In the same manner, MCF-7 cell growth was significantly increased by 3β-Adiol and genistein treatment, and these pro-proliferative effects appeared to be partially diminished following HSPB8 silencing. Therefore, based on our results, we conclude that HSPB8 positively modulates MCF-7 cell growth.

Notably HSPB8 also has an impact on cell migration. Indeed HSPB8 down-regulation combined with exposure to SERMs modulated the migratory ability of MCF-7 cells, since while 17β-estradiol increased migration, this pro-migratory effect was abolished by HSPB8 silencing. The pro-migratory activity of HSPB8 in cancer cells migration has also been described in ovarian cancer (SKOV3.ip1) cells in which it was demonstrated that HSPB8 acts as a positive regulator in TGF-β -induced migration of ovarian cancer cells and directs ovarian cancer toward progression; as expected, HSPB8 silencing had the opposite effects in these cells [42]. In our study we found that 3β-Adiol exerted an anti-migratory effect on MCF-7 cells maintained by HSPB8 silencing. Thus, similarly to PC cells [6, 35, 43], also in BC cells, estradiol and 3β-Adiol act as physiological antagonists on cell migration and differentially synergize with HSPB8. Omoto and Iwase [44] argued that the anti-migratory effects of 3β-Adiol are due to a preferential interaction of 3β-Adiol with ERβ that determines an inhibition of BC cell migration [43]. Also genistein exerted an anti-migratory activity, but in this case HSPB8 silencing did not influence the overall effect of genistein on cell migration. Notably, while some studies argue that genistein in BC cells is able to induce apoptosis, inhibits cell differentiation and inhibits angiogenesis [45–48], others suggest that genistein increases the metabolic activity in MCF-7 cells, thus enhancing growth and development of the tumor and ability to generate metastases [49, 50]. Based on preclinical data, Kwon [51] suggested that high concentration of genistein effectively inhibited the growth of BC cells regardless of their ER status. However, genistein at low concentration oppositely stimulated ER-positive breast tumors [51], and thus the potential of genistein in BC remains still largely debated.

In conclusion, the data here reported suggest that HSPB8 is involved in the mechanisms that regulate cell cycle and in those involved in cell migration. Although the data reported in this study provide the basis for advancing concrete hypotheses about the role played by HSPB8 in BC, further studies are needed to better clarify the molecular mechanisms that underlie its activities and the possibility of its eventual use as a therapeutic target.

## MATERIALS AND METHODS

### Cell culture and treatments

MCF-7, MDA-MB-231, T47D, DU145, PC3 and HepG2 cell lines were originally obtained from the American Type Culture Collection (Rockville, MD). Cells were routinely grown in RPMI 1640 medium (Biochrom KG, Berlin, Germany), supplemented with 5% foetal bovine serum (FBS) that was obtained from GIBCO (GIBCO, BRL, Grand Island, NY), glutamine (1mM) and antibiotics (100 IU/ml, penicillin G sodium and 100 μg/ml streptomycin sulphate) in a humidified atmosphere of 5% CO_2_, 95% air at 37°C.

HepG2 cells were cultured in MEM medium supplemented with 10% FBS, glutamine and antibiotics as described above.

The human BLM (NRAS-mutant, BRAF-wild type) melanoma cell line was provided by Dr. G.N. van Muijen (Department of Pathology, Radbound University Nijmegen Medical Center, Nijmegen, The Netherlands). This cell line is a subline of BRO melanoma cells isolated from lung metastases after subcutaneous inoculation of nude mice with BRO cells [52]. BLM cells were routinely cultured in DMEM medium supplemented with 10% FBS, glutamine and antibiotics as described above.

iPSC cells were obtained from fibroblast of healthy donor (P6-2 clone) and generated by Oct4, Klf4, Sox2 and c-Myc transfection. They were kindly provided by Dr. Christopher Grunseich and Dr. Kennet Fischbeck, NIH, Bethesda, MD, USA [53]. iPSC cells were cultured in Essential 8 medium (Life Technologies).

In all the experiments MCF-7, T47D and MDA-MB-231 cells were grown in RPMI medium supplemented with 5% FBS. At the starting of the experiment, for synchronizing cell growth, the medium was replaced with RPMI 1640 without FBS and without phenol red, in which the cells were kept overnight. During this time period, cell growth slowed down. The day after, the medium was one more time replaced with RPMI 1640 without phenol red containing 5% charcoal-treated FBS (E_2_-deficient medium).

### Reagents

17β-estradiol, estradiol valerate and genistein were from Sigma-Aldrich (St. Louis, MO, USA). 3β-Adiol was from Steraloids (London, UK); raloxifen and tamoxifen were kindly obtained from Siena Biotech (Siena, Italy).

### Plasmids and siRNA

The plasmid pCI-NEO-HSPB8 contains the sequence of human HSPB8 cloned in pCI (Promega, Madison, WI, USA) [54]; an empty vector was used as control. To silence endogenous HSPB8 expression, we used a custom siRNA duplex (CGG AAG AGC UGA UGG UAA AUU, Dharmacon, Thermo Scientific Life Sciences Research, Waltham, MA, USA); to silence endogenous HSPB1 we used a SMARTpool: ON-TARGETplus HSPB1 siRNA (L-005269-00-0005, Dharmacon, USA); a Non-Targeting siRNA (NT) was used as negative control (UAG CGA CUA AAC ACA UCA A, Catalog Item D-001210-01-05, Dharmacon, USA).

All plasmids were transfected using Jet-Prime Transfection Reagent and Jet-Prime Buffer (Kit Polyplus, Polyplus-transfection^®^ SA New York, NY, USA), according to the manufacturers' instructions.

### mRNA expression analysis

MCF-7 cells were seeded in 6-well plates at 300,000 cells/well, transfected and/or 1 day later treated for 2 days with 17β-estradiol (10nM), estradiol valerate (10nM), 3β-Adiol (1μM), genistein (1μM) raloxifen (1μM) and tamoxifen (1μM). 3 days after transfection, cells were harvested in 4M guanidium-isothiocyanate (containing 25mM sodium citrate pH 7.5, 0.5% sarcosyl and 0.1% 2-mercaptoethanol) and total RNA was isolated using phenol-chloroform extraction method [55]. RNA quantification was carried out by absorption at 260 nm. Total RNA (1μg) was treated with DNAse and reverse transcribed into cDNA using the High-Capacity cDNA Archive Kit (Applied Biosystems, Life Technologies Corporation) according to the manufacturer's protocol. Primers for real-time PCR recognising HSPB8, LC3, SQSTM1/p62, TFEB and RPLP0 mRNAs were previously described [56]. Primers for real-time PCR of the ERα, ERβ, ZK-SCAN3 and 18S were designed in accordance with recommendations accompanying the CFX 96 Real Time System (Bio-Rad, Hercules, CA, USA) on C-G base content and using the program Primer Express 3. Primers were synthetized by MWG Biotech (Ebersberg, Germany) with the following sequence: ERα upstream primer, 5′-CGGTTCCGCATGATGAACCT-3′ and ERα downstream primer, 5′- TGGTCCTTCTC TTCCAGAGACTTC-3′; ERβ upstream primer, 5′-AGCTGGCTGACAAGGAACTG-3′ and ERβ downstream primer, 5′- CAGGCTGAGCTCCACAA AGC-3′; ZK-SCAN3 upstream primer, 5′-GATGGAAAGCCAGTTGGAAA-3′ and ZK-SCAN3 downstream primer, 5′-AAATTCGGGTGAAGCCT TTT-3′ 18S upstream primer, 5′-GGATGTAAAGGAT GGAAAATACA-3′ and 18S downstream primer, 5′- TCCAGGTCTTCACGGAGCTTGTT -3′. The evaluated efficiency of each set of primers was close to 100% for target and reference genes. Real-Time PCR was performed using the CFX 96 Real Time System (Bio-Rad) in 10μL total volume, using the iTaq SYBR Green Supermix (Bio-Rad), and with 500nM primers. PCR cycling conditions were as follows: 94°C for 10 minutes, 35 cycles at 94°C for 15 seconds and 60°C for 1 minute. Melting curve analysis was always performed at the end of each PCR assay as a control for specificity. Data were expressed as Ct values and used for the relative quantification of targets using the ΔΔCt calculation. To exclude potential bias because of averaging, data were transformed using the equation 2^−ΔΔCt^ to give N-fold changes in gene expression; all statistics were performed with ΔCt values. Each sample was analyzed in triplicate (n=3); HSPB8, ERα, ERβ, LC3, SQSTM1/p62, TFEB and ZK-SCAN3 values were normalized with those of RPLP0.

### Western blotting analyses

MCF-7 cells were seeded in 6-well plates at 300,000 cells/well transfected and/or treated for 2 days with 17β-estradiol (10nM), estradiol valerate (10nM), 3β-Adiol (1μM), genistein (1μM) raloxifen (1μM) and tamoxifen (1μM). MCF-7 cells were harvested in RIPA buffer addicted with protease inhibitors, centrifuged, and washed in PBS. Protein concentration was determined using the Bradford assay. Equal amount of protein (20μg) were resolved on a 7-15% SDS-polyacrylamide gel electrophoresis (SDS-PAGE). Proteins were transferred using a transfer apparatus (BIO-RAD Trans Blot semi-dry). The membrane was washed with 10mM Tris-HCl, 150mM NaCl, 0.1% Tween 20 (TBST) for 30 min, blocked in TBST and 5% (w/v) dry skimmed milk, and then incubated with the primary antibody at 4°C o/n. For HSPB8, HSPB1, HSPB2, HSPB3, HSPB5, LC3, SQSTM1/p62, α-tubulin and actin detection we used a) rabbit polyclonal antibody against HSPB8 (kindly provided by Dr. Jacques Landry, Quebec, Canada; dilution 1:2000); b) rabbit polyclonal antibody against HSPB1 (kindly provided by Dr. Jacques Landry, Quebec, Canada; dilution 1:2000); c) mouse monoclonal antibody against HSPB2 (SC-136339 Santa Cruz Biotechnology; dilution 1:1000); rabbit polyclonal antibody aginst HSPB3 (SAB1100972 Sigma-Aldrich; dilution 1:1000); d) mouse monoclonal antibody against HSPB5 (SMC-159A STRESSMARQ; dilution 1:1000); e) rabbit polyclonal antibody against LC3 (L8918, Sigma-Aldrich; dilution 1:2500); f) rabbit polyclonal antibody against SQSTM1/p62 (ab91526, Abcam; dilution 1:1000); g) mouse monoclonal antibody against α-tubulin (T 6199, Sigma-Aldrich; dilution 1:4000); h) goat polyclonal antibody against actin (I-19, Santa Cruz Biotechnology; dilution 1:1000). Then, the membranes were washed and incubated for 1 hr at room temperature with secondary antibody conjugated with peroxidase (anti rabbit for HSPB8, HSPB1, HSPB3, LC3 and SQSTM1/p62, dilution 1:10,000; anti-goat for actin, dilution 1:5000; anti-mouse for HSPB2, HSPB5, dilution 1:5000 and α-tubulin, dilution 1:20.000). Immunoreactive bands were visualized using the enhanced chemiluminescence’s detection kit reagents (Clarity^TM^ Western ECL Substrate, Bio-Rad, Italy). A ChemiDoc XRS System (Bio-Rad) was used for the image acquisition.

### Immunofluorescence

MCF-7 cells were plated on 13mm diameter coverslips at 40.000 cells/well transfected and/or treated with 17β-estradiol (10nM), 3β-Adiol (10μM), and genistein (1μM). 2 days after treatments, cells were fixed in 4% paraformaldehyde (PFA), permeabilized in 0.5% Triton X-100 and then, the unspecific sites were blocked in 5% fetal bovine serum (Gibco) in PBS. Subsequently, cells were incubated with the primary antibodies o/n at 4°C. The primary antibodies used were: rabbit polyclonal anti-HSPB8 antibody (kindly provided by Jacques Landry, Quebec, Canada) 1:200; mouse monoclonal anti-α-tubulin antibody (Sigma-Aldrich) 1:200. Secondary antibodies (Rockland) were used at the concentration of 1:500 for 1 hr at room temperature: 611-700-127 (red, anti-rabbit) for the detection of HSPB8 and 710-702-124 (green, anti-mouse) for the detection of α-tubulin. The manufacturer guarantees the use of these secondary antibodies for multiple labelling procedures, since they are purified against cross-reactivity to other species. Nuclei were stained with DAPI (Sigma-Aldrich). Finally, the cells were washed in PBS before mounting using Mowiol 40-88 (Sigma-Aldrich). Images were collected using the UIC-Metavue 6.2.2 (UIC-Crisel Instr. Rome) imaging system on an Axiovert Zeiss 200 microscope, utilising a 40× magnification (NA 0.8) objective.

### Cell growth studies

Cells growth/viability was measured by MTT [3-(4,5-dimethylthiazol-2-yl)-2,5-diphenyltetrazolium bromide] assay. Briefly, culture medium was replaced with fresh medium containing MTT (1.5 mg/ ml) and the multiwells were incubated at 37°C for 1 hr, then the medium was removed and 2-propanol (500μl) was added to solubilize the crystals. The absorbance was red at 550 nm with an Enspire 2300 Multimode Plate Reader (Perkin Elmer, Italy).

To study the effect of SERMs on MCF-7 and MDA-MD-231, cells were seeded in 24-well plates at 40,000 cells/well, transfected and/or treated for 2 days with 17β-estradiol (10nM), estradiol valerate (10nM), 3β-Adiol (1μM), genistein (1μM) raloxifen (1μM) and tamoxifen (1μM); cell viability was analyzed as described above.

### FACScan analysis

For FACScan analysis the adherent MCF-7 and MDA-MB-231 cells were seeded in 12-well plates at 80,000 cells/well. After 24 hrs, cells were transfected with 0.5μg of empty vector, 0.5μg of pCI-NEO-HSPB8 plasmid, 40nM of siRNA-HSPB8 and 50nM of siRNA-HSPB1 (only for MCF-7 cells); 3 days after transfection, cells were washed in PBS, harvested, centrifuged, and re-suspended in 400μl of Permealization buffer (100mM Tris pH 7.4, 150μM NaCl, 1μM CaCl_2_, 0.5μM MgCl_2_, 0.1% NP-40) addicted with propidium iodide 5μM. Cells were incubated at room temperature for 15 min. Cells were then measured on a FACScan flow cytometer (FACS Calibur - BD Biosciences, San Jose, CA, USA). Cytofluorimetric results were analyzed using CellQuest (BD Biosciences) analysis software. All the flow cytometric measurements were done using the same instrument settings, and at least 10,000 cells were measured in each sample. The results of three separate experiments are presented as the mean ± SD. Each experimental group was performed in triplicate.

### Haptotaxis assay

Briefly, cell migration assay was performed using a 48 well-Boyden chamber (NeuroProbe, Inc., Gaithersburg, MD, USA) containing 8μm polycarbonate filters (Nucleopore, Concorezzo, Milan, Italy). Filters were coated on one side with 50μg/ml laminin, rinsed once with PBS, and then placed in contact with the lower chamber containing RPMI 1640 medium. MCF-7 cells, treated with 17β-estradiol (10nM), 3β-Adiol (1μM) and genistein (1μM) for 2 days after transfection with siRNA-HSPB8, were collected and then added in aliquots (75,000 cells/50μl) to the top of each chamber and allowed to migrate through coated filters for 4 hrs. At the end of the incubation, the migrated cells attached on the lower membrane surfaces were fixed, stained with Diffquik (Biomap, Italy) and counted in standard optical microscopy.

The results of four separate experiments of migration are presented as the mean ± SD. Each experimental group consisted of 12 samples. The results are expressed as a percentage of migrated cells *versus* control cells.

### Statistical analysis

Statistical analysis was performed by one-way ANOVA followed by Bonferroni multiple comparison tests. *p<0.05 was considered statistically significant.

## SUPPLEMENTARY FIGURE


