# Supplementary material for: The small heat shock protein B8 (HSPB8) modulates proliferation and migration of breast cancer cells
Source: Oncotarget. 2017 Jan 2;8(6):10400–15. doi: 10.18632/oncotarget.14422 (PMC5354667; doi:10.18632/oncotarget.14422)
Supplement: Supplementary file 1 [file oncotarget-08-10400-s001.pdf]

The small heat shock protein B8 (HSPB8) modulates proliferation and migration of breast cancer cells

SUPPLEMENTARY FIGURE

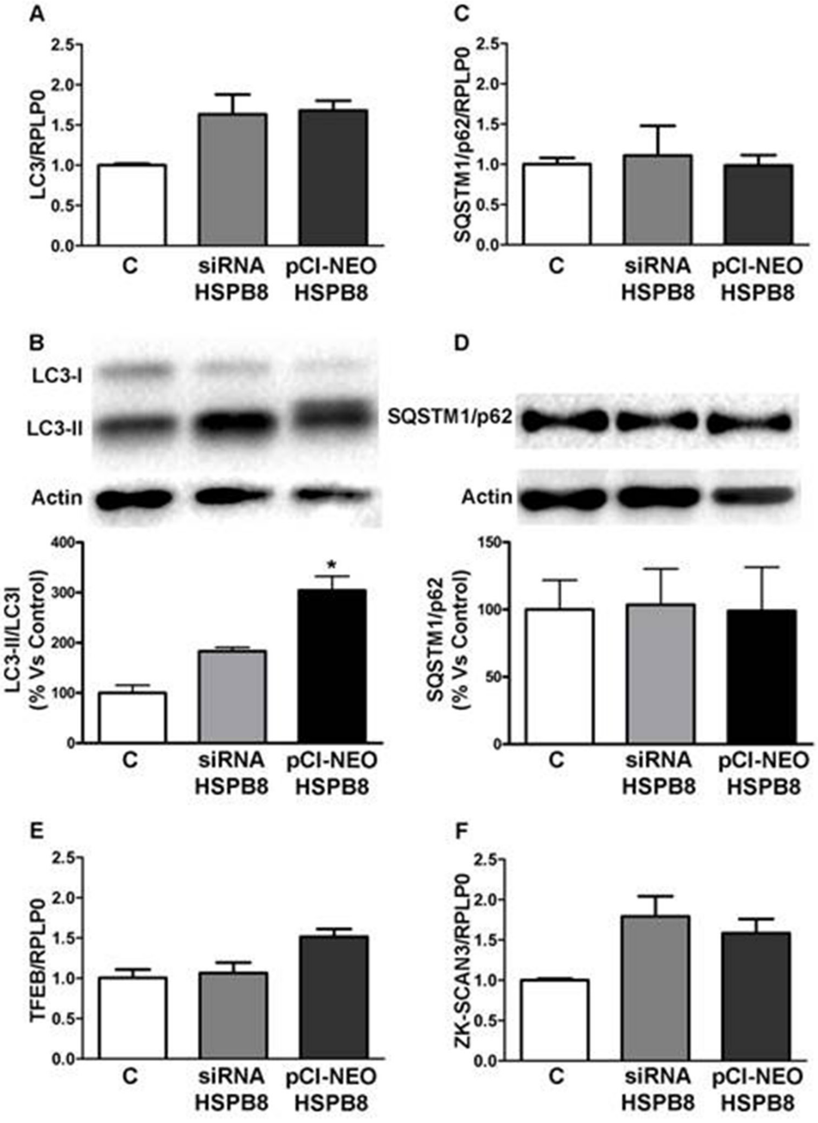

Supplementary Figure 1: Expression of LC3, SQSTM1/p62, TFEB and ZK-SCAN3 in MCF-7 cells transfected with siRNA-HSPB8 or pCI-NEO-HSPB8. Cells were transfected either with pCI-NEO-HSPB8 plasmid or with siRNA-HSPB8 and the analyses was performed 3 days after transfection. LC3 **A, B.** and SQSTM1/p62 **C, D.** mRNA and protein levels were measured by real-time RT-PCR analysis and Western blot analysis. TFEB **E.** and ZK-SCAN3 **F.** mRNA levels were quantified by real-time RT-PCR analysis. *$p<0.05$ *vs* Control. There were no significant differences in LC3 mRNA levels neither after up- nor down-regulation of HSPB8, although we observed a trend towards increase. On the contrary, western blot analysis revealed that both down- and up-regulation of HSPB8 increased LC3-II levels as compared to untreated control cells; however, this increase was significant only after HSPB8 up-regulation. There were no significant differences in SQSTM1/p62 mRNA and protein levels neither after down- nor after up-regulation of HSPB8. Similarly, neither the expression of TFEB, nor that of ZK-SCAN3 were modified by HSPB8 modulation.
